# Relationship between hope, optimism, emotional regulation, and pain in fibromyalgia

**DOI:** 10.1007/s10067-025-07742-z

**Published:** 2025-10-17

**Authors:** Avani Patel, Jeeshma Vijin, Abhishek Patil

**Affiliations:** 1 Department of Clinical Psychology, Manipal College of Health Professions, Bengaluru, Manipal Academy of Higher Education, Manipal, Karnataka India; 2 Department of Rheumatology and Clinical Immunology, Manipal Hospitals, Bengaluru, India

**Keywords:** Emotional regulation, Fibromyalgia, Hope, Optimism, Pain

## Abstract

**Background:**

Fibromyalgia (FM) is a chronic pain disorder that is marked by psychological distress. While previous research has predominantly focused on negative outcomes such as anxiety and depression, this study investigates the role of positive psychological constructs—hope, optimism, and emotional regulation—in relation to pain perception among individuals with FM.

**Objectives:**

The study aims to find the relationship between hope, optimism, emotional regulation, and pain in patients with fibromyalgia.

**Methods:**

A total of 270 patients with FM were included in the study. The present study used the Adult Hope Scale, Life Orientation Test—Revised, Emotional Regulation Questionnaire, and Fibromyalgia Impact Questionnaire Revised to measure hope, optimism, emotional regulation, and pain, respectively. Spearman’s correlations were used to explore the relationship between psychological variables and pain severity.

**Results:**

Hope showed a moderate negative correlation with pain, *r* =  − .34, 95% CI [− .44, − .23], *p* < .001, indicating that individuals with higher levels of hope experienced lower pain. The study found a weak negative correlation of optimism with pain, *r* =  − .12, 95% CI [− .15, − .09], *p* = .042.

**Conclusion:**

These findings suggest that hope plays a pivotal role in modulating pain experiences in FM and should be considered in treatment planning. The study emphasizes the need for multidisciplinary care that integrates cognitive-behavioral techniques, emotion regulation training, and resilience-building exercises for symptom management and overall wellbeing of the patient with chronic pain. The study accounted for age and gender in the analysis; however, other confounding variables such as socioeconomic status, education, disease duration, and comorbidities like depression, anxiety, and social and relational factors were not measured. These factors may influence both psychological outcomes and pain perception.

**Trial registration:**

The trial was registered in the clinical trial registry in India. CTRI Registration CTRI/2025/02/081217 (Registered on: 24/02/2025). The link of the trial registry is https://ctri.nic.in/

**Table:** 

**Key Points**
• *Hope and optimism may both serve as psychological resources that help regulate the severity of pain*. • *Pain outcomes may not be shaped by emotional regulation alone*. • *Routine assessment of hope and optimism may add value to fibromyalgia management protocols*.

## Introduction

Fibromyalgia (FM) is characterized by persistent, widespread pain and a variety of symptoms, including dysfunctions in cognition. It is more common in women than in men, but in clinical settings—particularly tertiary care settings—the sex-based difference is considerably more pronounced than in population-based samples [1]. The prevalence rate of FM in a neurology outpatient setting in India was reported as 13.9%, with 942 out of 6784 screened patients diagnosed with FM [2]. Similarly, a study has found that approximately 10% of individuals with inflammatory bowel disease (IBD) met criteria for fibromyalgia, highlighting comorbidity within that specific patient group rather than population-wide prevalence [3].

Individuals with FM often struggle to meet work-related expectations due to chronic symptoms such as functional impairment, which can increase the need for rest, reduce productivity, and contribute to emotional distress. The condition is closely associated with negative affect, maladaptive emotional responses—including anger suppression, outward expression of anger, rumination, and pain catastrophizing—and cognitive difficulties [4].

Despite the growing recognition of these psychological dimensions, research has only recently begun to explore positive individual factors, such as hope, that may serve as psychological buffers against the challenges posed by chronic illnesses like FM. Considering the high levels of emotional exhaustion and stress experienced by this population, examining the role of hope along with other psychological factors may offer valuable insights into resilience-building and symptom management [5].

Individuals with higher levels of hope are often better able to manage adversities because of increased psychological resilience. In contrast, disruptions to goal-directed thinking can heighten emotional and physical fatigue. Such disruptions, often caused by physical limitations or social stressors, particularly affect individuals with long-term health conditions [5].

Closely associated with hope is optimism, a personality trait characterized by a positive outlook on future events [6]. It was found that optimistic individuals are more likely to use problem-focused coping strategies, which in turn contribute to greater stress resilience and an overall improved sense of control over life’s difficulties [6]. In addition to hope and optimism, emotional regulation plays a critical role in psychological adaptation to chronic conditions.

Studies have also emphasized the importance of emotional regulation not only in clinical populations but also in high-stress environments like academia [7]. Their findings suggest that effective emotional regulation can act as a protective factor, improving overall functioning and buffering against the psychological demands associated with chronic pain [8, 9].

Although considerable attention has been given to the physical and psychological burdens of fibromyalgia, there is a limited number of studies examining protective psychological factors, such as hope, optimism, and emotional regulation, within the Indian context. Most existing literature has focused on the negative consequences of FM, often overlooking the potential strengths that individuals may use to cope with the condition. Understanding these relationships may inform clinical practices and interventions aimed at enhancing resilience, promoting well-being, and ultimately improving the quality of life for individuals with fibromyalgia. By assessing multiple constructs together (hope, optimism, and emotional regulation), it may provide a more comprehensive view of their collective impact on pain experiences and help in pain management and treatment.

## Methods

### Aim

To find the relationship between hope, optimism, emotional regulation, and pain in patients with fibromyalgia.

### Setting

The study was conducted in the Department of Rheumatology, Manipal Hospitals, Bangalore, India.

### Design

The present cross-sectional correlational study used a convenience sampling method to recruit participants. The sample size for the study was calculated to be 269 using the formula *N* = [(*Z*_α_ + *Z*_β_)/*C*]^2^ + 3. Using a two-tailed significance level of *α* = 0.05 (*Z*_1-_*α*/2 = 1.96), a power of 80% (*Z*_1-_*β* = 0.84), and an anticipated effect size of |*r*|= 0.17, *C* = 0.5 * ln[(1 + r)/(1-r)] based on previous literature [5].

### Recruitment and participants

Participants who were included in the study were diagnosed with FM according to the diagnostic criteria of ACR (2016). Individuals from 18 years of age to 60 years were included in the study. Individuals with other rheumatological disorders such as rheumatoid arthritis or pain conditions which are not related to fibromyalgia were excluded. There was no missing data in the study, and no participants were excluded after enrolling in the study.

### Procedure

The study was approved by the Institutional Research Committee (IRC) of the Manipal College of Health Professions, Manipal Academy of Higher Education, and by the Scientific and Ethical Committee of Manipal Hospitals. It was registered with the Clinical Trials Registry of India (CTRI; Registration No. CTRI/2025/02/081217; Registered on: 24/02/2024) prior to the commencement of data collection. After registration, eligible participants were informed about the study and invited to participate. Written informed consent was obtained from those who agreed. Participants were assured that they could decline or withdraw from the study at any stage without providing a reason. The outcome measures were administered after obtaining consent from the participants.

### Variables

Data was collected with a five-part questionnaire which included socio-demographic details and the following scales:

The Adult Hope Scale (AHS)—developed by C.R. Snyder and colleagues (1991)—is used to measure the level of hope in adults. The Adult Hope Scale is based on hope theory. It has 12 items and has direct scoring, on the scale of 1 to 8. The scores 0 to 39 indicate low hope, 40 to 47 indicate hopeful, 48 to 55 indicate moderately hopeful, and scores above 56 indicate highly hopeful. The scale exhibited strong internal consistency (Cronbach’s alpha = 0.88), reliable test–retest reliability (ICC = 0.76), and robust validity, as evidenced by lower correlations with depression and higher correlations with day-to-day functioning and activity levels [10].

The Life Orientation Test-Revised (LOT-R)—developed by Michael F. Scheier and Charles S. Carver (2002)—assesses individuals’ degrees of optimism vs pessimism, which influences mental and physical well-being. LOT-R is a typical psychological tool that measures one’s dispositional level of optimism, offering useful information about potential therapies, such as those aimed at addressing problematic thinking patterns. After reverse scoring of selected items, a total score of 0 to 13 indicates low optimism (or higher pessimism); 14 to 18 indicates moderate optimism, and 19 to 24 indicates high optimism. LOT-R has shown good reliability (Cronbach’s alpha = 0.80) and validity (ranging from 0.34 to 0.65) in a population of healthy adults [11].

Emotional Regulation Questionnaire (ERQ)—developed by James J. Gross and Oliver P. John (2003)—measures a person’s emotional regulation. The 10-item test assesses respondents’ ability to manage their emotions through Cognitive Reappraisal and Expressive Suppression. Each question is rated on a 7-point Likert scale, with 1 indicating strong disagreement and 7 indicating strong agreement. Items 1, 3, 5, 7, 8, and 10 are scored for emotional reappraisal, and items 2, 4, 6, and 9 are scored for emotional suppression. Higher emotional reappraisal indicates an individual’s flexibility in reframing situations to manage emotions, generally linked to better psychological health, lower depression/anxiety, and higher social functioning. Low emotional reappraisal and higher emotional suppression indicate that the individual rarely interprets situations and hides feelings and are generally linked to greater negative affect, stress, and interpersonal strain. High scores on both facets indicate that individuals use various strategies in dealing with their emotions, whereas low scores on both facets indicate limited use of either of the emotional regulation strategies. The reliability of the test is 0.76, and it shows good criterion validity and internal validity [12].

Fibromyalgia Impact Questionnaire – Revised (FIQR)—developed by Robert M. Bennett and colleagues (2009)—gives insight into the pain experienced due to FM. It shows good content and discriminant validity. The items on the test were tested for their reliability as well. It has three domains that are scored separately. The sum of the scores of the three domains is then interpreted to find the impact of the illness. Higher scores indicate greater symptom severity and functional impairment. The FIQR shows good reliability with a correlation coefficient of 0.83 (*p* < 0.005), along with good convergent and discriminant validity [13]. Pain severity was measured using a visual analogue scale of 0 (no pain) to 10 (unbearable pain).

### Analysis

Jamovi (version 2.6.44) was used for data analysis [14].

Descriptive statistics summarized demographic and clinical variables. Mean and standard deviation for the demographic variables were calculated. Normality was verified via the Shapiro–Wilk test. Spearman’s correlation was used for the sample size (*n* = 270) to assess the relationship between hope and pain, optimism and pain, and emotional regulation and pain.

## Results

### Descriptives of sociodemographic and patient variables

The study included 270 eligible participants for the study aged 18 to 60 years. The mean age of the participants was 42.27 ± 10.59 years. The participants included more females (234, 86.7%) than males (36, 13.3%) in the study and sex distribution was 26 (13.3%) males and 234 (86.7%) females. There was no missing data in the study, and no participants were excluded after enrolling in the study. The patient variables are explained in Table 1.

**Table 1:** Descriptives of sociodemographic and patient variables

Descriptives	Mean (SD)/*N* %
Age	42.27 (10.59)
Gender—male	36 (13.3)
Gender—female	234 (86.7)

### Relationship between the variables

Spearman’s correlation was computed to assess the associations between pain and psychological variables—hope, optimism, and emotional regulation (Table 2). Hope showed a moderate negative correlation with pain, *r* =  − 0.34, 95% CI [− 0.44, − 0.23], *p* < 0.001, indicating that individuals with higher levels of hope experienced lower pain. The study found a weak negative correlation of optimism with pain, *r* =  − 0.12, 95% CI [− 0.15, − 0.09], *p* = 0.042.

**Table 2:** Correlation between the variables pain, hope, optimism, and emotional regulation

Variable	Pain	Hope	Optimism
Hope	− 0.339***	—	
Optimism	− 0.124*	0.270***	—
Emotional regulation	− 0.027	0.332***	0.129*

*p < 0.05, ***p < 0.001

## Discussion

This study confirms that positive psychological factors—hope and optimism—are significantly associated with lower pain severity in fibromyalgia, independent of emotional regulation capacity, in an Indian cohort. The study comprised 270 participants aged between 18 and 60 years (mean = 42.27, SD = 10.59). The gender distribution was notably skewed, with 86.67% identifying as female and 13.33% as male. This demographic pattern is consistent with epidemiological studies that indicate a higher prevalence of FM among women, potentially due to biological, hormonal, or psychosocial factors contributing to pain sensitivity and reporting behavior [1].

This finding aligns with the prior research [5] suggesting that higher levels of hope may reduce the perception of pain, likely due to stronger goal-setting abilities, enhanced coping mechanisms, and a proactive mindset.

Optimism also demonstrated a statistically significant but weak negative correlation with pain. Although this suggests that a positive outlook may be associated with reduced pain perception, the correlation strength is modest. Previous studies [6, 8] have shown that optimism contributes to psychological resilience and stress buffering, but the present findings indicate it may play a more supportive rather than central role in pain modulation.

In contrast, emotional regulation did not show a significant correlation with pain. This can be explained by the Common-Sense Model of Illness Self-Regulation (CSM), which suggests that pain outcomes are not directly shaped by emotion regulation alone, but rather involve a complex interplay among illness perceptions, coping strategies, and emotional responses [15]. Research on emotional regulation and illness adaptation conducted in women with breast cancer indicates that effective adjustment involves proactive coping, minimal avoidance and emotional suppression, positive cognitive changes, and response modulation activities. Hence, patients who use adaptive coping strategies such as seeking information, adherence to treatment, and modifying health behavior may have better emotional regulation, which explains the non-significant association observed in the study [16]. Also, the study was conducted in India, so the cultural factors like collectivism and family support may influence emotional regulation, hope, and optimism. Moreover, EPQ may not identify the culturally specific emotional regulation strategies used by individuals with chronic pain in the Indian context. Hence, we recommend future studies to use culturally adapted measures to understand emotional regulation in pain perception.

## Strengths and limitations

One of the major strengths of this study is its focus on positive psychological variables—namely hope, optimism, and emotional regulation, understanding pain perception among individuals with fibromyalgia (FM). While previous research has largely emphasized pathological aspects such as distress and disability, this study contributes to a strength-based approach, highlighting psychological resources that may buffer the impact of chronic pain.

The statistical approach employed provided relational insights into how psychological variables interact with pain.

Additionally, the study addresses a relatively under-researched area in the Indian context, helping bridge cultural gaps in FM research and creating opportunities for culturally relevant therapeutic applications.

A few limitations of this study include the cross-sectional design of the study limits causal inference. It remains unclear whether higher hope reduces pain, or if individuals with lower pain levels are simply more hopeful. The study accounted for age and gender in the analysis; however, other confounding variables such as socioeconomic status, education, disease duration, and comorbidities like depression, anxiety, and social and relational factors were not measured. These factors may influence both psychological outcomes and pain perception. Future studies should aim to include these variables to provide a more comprehensive understanding of the findings.

## Future recommendations

Future research should explore the mediating and moderating roles of emotional regulation and optimism in the relationship between hope and pain. Longitudinal studies are needed to provide clarity on the directionality of these relationships, and intervention-based designs would help to assess the causal impact and effectiveness of hope-based interventions. Additionally, qualitative approaches could offer richer insights into how individuals with FM conceptualize and use psychological strengths in managing their condition.

## Implications and conclusion

The findings of this study support incorporating psychological assessments and hope-enhancing interventions into pain management programs, such as goal-setting workshops, solution-focused therapy, and cognitive-behavioral strategies aimed at increasing agency and perceived pathways. Hope is considered a malleable trait that can be cultivated through psychological interventions, making it a practical and impactful target in clinical settings.

While optimism and emotional regulation did not emerge as strong independent predictors, their weak-to-moderate correlations with pain and with each other suggest that they may act as supportive or mediating variables. For example, individuals high in hope may naturally engage in more optimistic thinking and employ more effective emotion regulation strategies, contributing to greater resilience to pain.

These findings indicated the need for multidisciplinary care that integrates cognitive-behavioral techniques, emotion regulation training, and resilience-building exercises for symptom management and overall well-being of the patient with chronic pain.

To conclude, this study explored the relationships between hope, optimism, emotional regulation, and pain in individuals with fibromyalgia. Among the variables examined, hope was found to have a moderate correlation with pain. Optimism showed a weak but significant correlation with pain, whereas emotional regulation, while theoretically relevant, did not show a significant relationship with pain in this study. The findings reinforce the need for integrated care models that prioritize not just symptom management but also psychological empowerment. By targeting hope through structured interventions, clinicians may be able to mitigate pain experiences, enhance coping, and improve overall quality of life for FM patients.

## Data Availability

The datasets used and/or analyzed during the current study are available from the corresponding author on reasonable request.

## Abbreviations

ACR: American College of Rheumatology
AHS: Adult Hope Scale
ERQ: Emotional Regulation Questionnaire
FIQR: Fibromyalgia Impact Questionnaire
FM: Fibromyalgia
LOT-R: Life Orientation Test – Revised
